# Protocol for a randomized controlled trial exploring the brain mechanism and therapeutic effect of electroacupuncture on cognitive function and sleep quality in chronic insomnia

**DOI:** 10.1186/s12906-023-04242-y

**Published:** 2023-11-08

**Authors:** Linhao Lu, Lizhen Liao, Jiaorong Zheng, Weiyi Lin, TaiShun Wang, Xiuyun Wen

**Affiliations:** 1 School of Health Science, Guangdong Pharmaceutical University, Guangzhou, 51000 China; 2 Guangdong Provincial Engineering and Technology Research Center of Light and Health, Guangzhou, 51000 China

**Keywords:** Electroacupuncture, Chronic insomnia, Clinical trial, Randomized controlled trial, Brain mechanism

## Abstract

**Background:**

Insomnia is a prevalent sleep disorder that affects up to 15% of the population worldwide and is the second most common mental health issue. There is increasing interest in the effects of long-term insomnia on cognitive function. Electroacupuncture can effectively improve cognitive function and sleep quality, yet the underlying brain network mechanisms remain unclear. This study aims to explore the network regulatory mechanisms associated with enhanced cognitive function and sleep quality, providing theoretical support for the use of electroacupuncture in the clinical treatment of chronic insomnia.

**Methods:**

This study is divided into two parts. Sixteen individuals with chronic insomnia and 16 healthy controls of similar age and gender will be recruited in Study 1 to examine the brain network topology of individuals with chronic insomnia. Study 2 will be a randomized controlled trial with 120 chronic insomnia patients divided into three groups: Group A (electroacupuncture plus placebo drug), Group B (drug plus placebo electroacupuncture), and Group C (placebo electroacupuncture plus placebo drug). Participants will be exposed to 24 treatments over an 8-week period (3 times per week) and monitored for 12 additional weeks. The primary outcome measure will be changes in brainwave data from before to after the treatment. In addition, the Wisconsin Card Sorting Test and the Pittsburgh Sleep Quality Index will be utilized as secondary outcomes to measure from before to after treatment and during the follow-up. A correlation analysis will be conducted to explore links among modifications in brainwave patterns, Wisconsin Card Sorting Test scores, and Pittsburgh Sleep Quality Index scores. Additionally, any adverse events will be strictly monitored.

**Discussion:**

Electroacupuncture may represent an alternative treatment for chronic insomnia, and this trial is expected to reveal the brain mechanism by which electroacupuncture improves cognitive function and sleep quality in chronic insomnia patients.

**Trial registration:**

ChiCTR2200060150 (Chinese Clinical Trial Registry, http://www.chictr.org.cn, registered on 20 May 2022).

## Background

In modern society, individuals experience more stress, leading to higher prevalence rates of physical and mental illness. Chronic insomnia is one of the leading mental health issues worldwide, with a global prevalence rate of 10–15% [1, 2]. This disorder has considerable negative effects on the physical and mental well-being of those affected. It significantly impairs cognitive functioning, such as attention, memory, and alertness, leading to decreased learning capacity, reduced work productivity, and an increased risk of traffic accidents [3–5]. Elucidating cognitive changes in those with chronic insomnia is a crucial focus of clinical therapy.

Brain imaging studies have indicated that chronic insomnia patients exhibit changes in brain structure and function [6, 7]. Voxel-based morphometry analysis has revealed a decrease in gray matter volume in the dorsolateral prefrontal cortex of these patients [8]. Furthermore, studies have indicated changes in the functional connectivity between the frontal and parietal lobes in individuals with insomnia. There is a notable correlation between the functional connectivity between the left dorsolateral prefrontal cortex and the left inferior parietal lobule and scores on the self-report sleep quality questionnaire [9]. Experiments on sleep durations and sleep deprivation have revealed that the frontal lobe is more sensitive to these impacts, with abnormal activation occurring in the frontal lobe (such as in the DLPFC and MPFC) during cognitive-related tasks [10]. The dorsolateral prefrontal cortex is therefore a brain region commonly administered noninvasive stimulation to treat insomnia [11]. These findings suggest a connection between insomnia and abnormal frontal lobe function.

Acupuncture has been demonstrated to improve the sleep quality and cognitive functioning of those with insomnia [12–14]. Our previous studies also revealed that acupuncture can effectively enhance sleep quality in patients with insomnia and depression [15]. Nevertheless, the precise brain mechanisms of acupuncture have yet to be elucidated. We conducted a study on four individuals with insomnia to explore the immediate effects of acupuncture. The results showed that the functional network topology of the entire brain changed throughout the course of acupuncture, with a trend toward higher global and local efficiency than those of sham acupuncture. Notably, local efficiency in the dorsolateral prefrontal cortex was increased. Improving cognitive functioning and sleep quality may be linked to the restoration of brain network topology in patients with insomnia, and the dorsolateral prefrontal cortex may be a critical network node.

Graph theory analysis of brain networks has the advantage of allowing simultaneous analysis of the functional connectivity patterns among all brain areas, and it can also be used to investigate the organization of brain networks in response to various interventions, which may reveal the brain mechanism by which acupuncture intervention alleviates chronic insomnia. By using EEG, source localization and graph theory-based brain network analysis methods, this study aims to investigate the effectiveness of acupuncture in insomnia. It will help understand reveal the changes in brain network characteristics in patients with chronic insomnias, evaluate network changes after acupuncture treatment, and explore the correlation between these changes and improved cognitive function and sleep quality. This research has the potential to provide a theoretical basis for treating chronic insomnia in clinical practice, thereby improving the quality of life of this population.

## Methods

### Trial design and setting

**Fig. 1:**
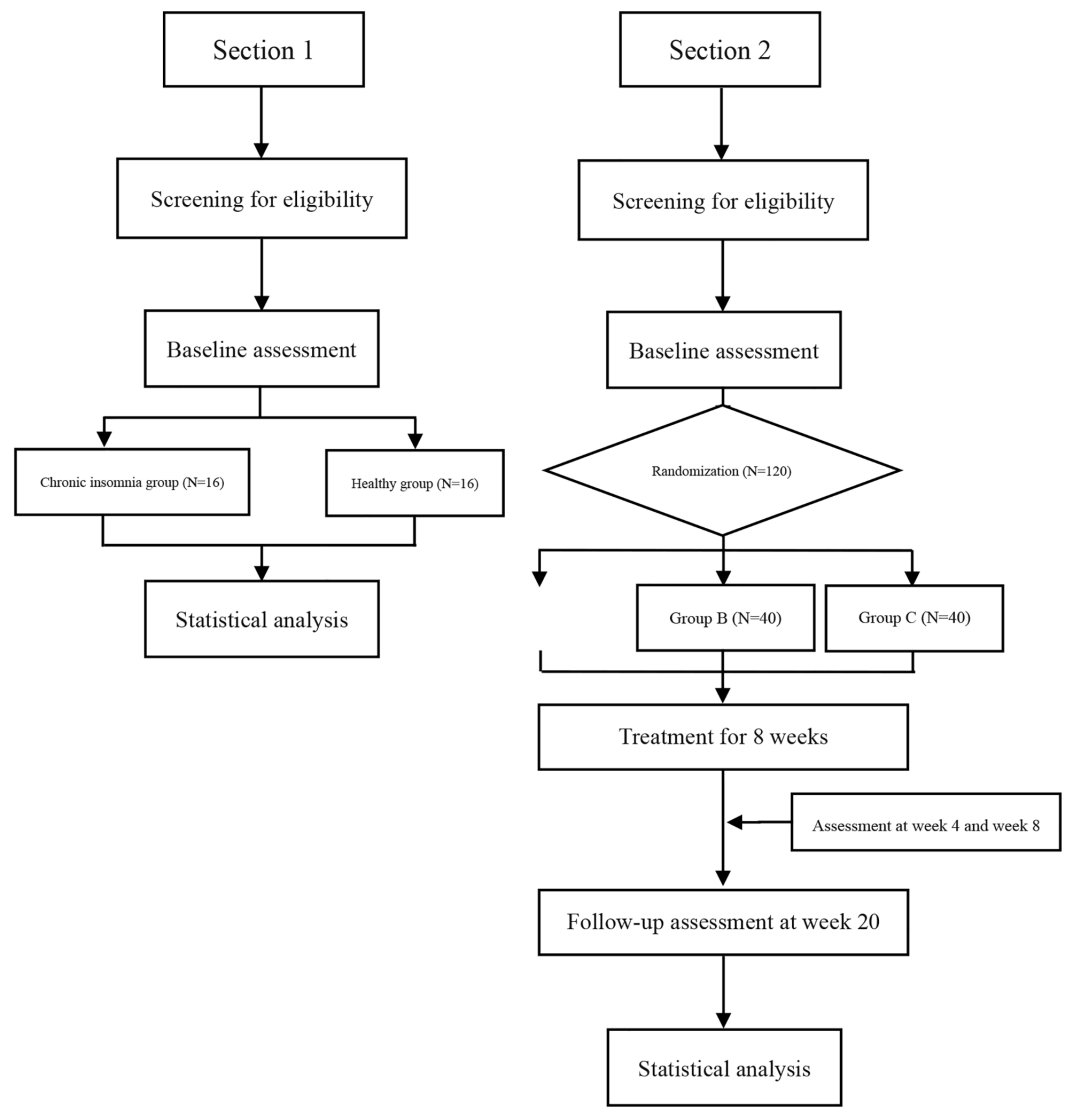
Flow chart of the trial procedure

The study is composed of two parts. Study 1 will recruit 16 individuals with chronic insomnia and 16 corresponding healthy controls, comparing changes in brain network topological characteristics between the two groups. Study 2 will be a randomized, double-blind controlled trial comprising 8 weeks of treatment and up to 12 weeks of follow-up. The experimental procedure is shown in Fig. 1.

This protocol was developed accordance with the Standard Protocol Items: Recommendations for Interventional Trials [16].

### Patient and public involvement

No patients have been involved.

### Patients

Participants with chronic insomnia will be recruited through posters (produced by the Publicity Department of the First Affiliated Hospital of Guangdong Pharmaceutical University), WeChat, and official WeChat accounts. The researchers will use the American Psychiatric Association DSM-5 criteria for insomnia to assign a diagnosis. Individuals who meet the inclusion criteria will receive free complimentary clinical testing and treatment to encourage enrollment. Before random allocation into groups, written informed consent will be obtained from eligible patients.

### Inclusion criteria

#### Study 1

The eligibility criteria for participants with chronic insomnia in Study 1 will be as follows: meet the diagnostic criteria for insomnia (depressive disorder due to another medical condition) as outlined in the DSM-5, have not received any systematic treatment for insomnia within the three months prior to the study, have a PSQI score of 8 points or higher, have a HAMD score of less than 7 points, and have a HAMA score of less than 14 points. Additionally, individuals must have had insomnia for at least 3 months, be between the ages of 18 and 50 years, be right-handed, and provide voluntary consent to participate in the study. Individuals between the ages of 18 and 50 years who have no sleep problems, have a PSQI score of 8 or less, have a HAMD score of 7 or less, have a HAMA score of 14 or less, have undergone a physical exam, are right-handed and have consented to participate in the study by signing the informed consent form will be considered eligible healthy controls.

#### Study 2

The inclusion criteria for patients with chronic insomnia in Study 2 will be the same as those in Study 1.

### Exclusion criteria

The exclusion criteria will be as follows: (1) severe mental illnesses such as depression, anxiety, or schizophrenia; (2) suicidal tendencies; (3) other severe systemic diseases that require treatment; (4) sleep apnea; or (5) a history of head trauma. In Study 2, in addition to the above exclusion criteria, subjects who are afraid of acupuncture and those with skin lesions at the puncture site will also be excluded.

### Randomization and blinding

The “Proc plan” feature of SAS 9.3(SAS Institute Inc., Cary, NC, USA) will be employed to create the necessary random allocation plan for the study. Eligible subjects will be assigned to Group A (electroacupuncture plus placebo drug), Group B (drug plus placebo electroacupuncture), or Group C (placebo electroacupuncture plus placebo drug) at a ratio of 1:1:1. All patients will be seen by appointment. During the acupuncture intervention, which will be completed in a quiet, separate treatment room to avoid contact and communication between patients in different groups, the therapist will instruct the patient to close their eyes. Only the acupuncturist performing the needle insertion throughout the trial will be aware of the patient’s group allocation. The subjects, evaluators of therapeutic effects, and individuals conducting the statistical analysts will be blinded to group allocation.

### Intervention

The treatment in this project will be administered by a professional acupuncturist with more than 3 years of experience. Treatment will be administered three times a week, with a minimum of 24 h between each session, over 8 weeks (24 treatment sessions).

An SDZ-V type Hua Tuo brand electronic acupuncture treatment instrument and needles (0.30 × 25 mm) will be used in this trial. Both the needles and the electroacupuncture device will be purchased from Suzhou Medical Supplies Factory Co., Ltd. (Production Enterprise License: Su Shi Yao Jian Xie Chan Xu 2001-0020; Registration Certificate Number: Su Shi Yao Jian Xie (Zhun) Zi 2004 No. 2,270,202).

### Electroacupuncture

The research team has previously demonstrated the effectiveness of acupuncture of the bilateral LU7 and KI6, DU20 and EX-HN3 in treating insomnia; thus, these acupoints will be the primary sites selected in this study, and their locations will be identified according to the WHO standard acupuncture locations [17].

The patient will lie down in a supine position. After the skin is disinfected, sterile adhesive pads will be affixed to the acupoints. Needles at LU7 and KI6 will be inserted to a depth of 0.3–0.5 inches. Needles at DU20 and EX-HN3 will be inserted at an angle of 30 degrees toward the back of the head and the tip of the nose, respectively, at a depth of 0.5 inches. The needles should be carefully lifted, inserted, and rotated three times until the patient feels achiness, heaviness, and numbness (known as de qi) at all acupoints. After 30 min, the needles will be removed.

Electroacupuncture will be administered using a sparse-dense wave with a frequency of 10/50 Hz and a current intensity of 0.5-1.0 mA, adjusted according to the patient’s tolerance.

### Sham electroacupuncture

Participants in the control group will receive a sham acupuncture treatment at the same acupoints. After disinfecting the skin, adhesive pads will be placed on the acupoints, and placebo needles will be placed into the pads without penetrating the skin. The same procedures and treatment parameters as used in the acupuncture group will be followed. The patient will be informed that a mild electric current is being administered, although they may not feel it.

### Drug

For a continuous treatment period of 8 weeks, 10 mg of zopiclone hydrochloride tablets will be taken orally before bedtime every day. The drug registration number is H20090001, the import permit number for psychotropic drugs is TPI20090302 and the approval number for imported drug packaging is National Drug Approval J20090050.

### Placebo drug

A placebo drug composed of starch will be administered with the same appearance, administration method, and treatment duration as the positive control drug.

### Follow-up

To evaluate the sustainability of the effects, a follow-up assessment will be conducted in the 20th week after the completion of baseline data collection.

### Outcomes

#### Primary outcomes

EEG data will be the primary outcome measure and recorded before and after the treatment. During EEG recording, subjects will be in a quiet room and wear a 64-lead Waveguard Original (CA-208, ANT neuroo, the Netherlands) Ag/AgCl EEG cap, keeping their eyes closed. EEG data will be collected with an electrode distribution according to the international 10–20 standard. EEG recordings will be performed with the company’s eego64 acquisition software, version 1.8.2, with the CPz and AFz electrodes used as the reference and ground electrodes, respectively. The sampling frequency will be 1000 Hz, and the electrode impedance will be reduced to less than 5 kΩ for each recording.

The preprocessed data will be transformed into a binary matrix to measure brain network parameters, including global efficiency, local efficiency, node degree, and node betweenness centrality.

Each index will be calculated as follows:

- Global efficiency

$${E}_{\text{g}\text{l}\text{o}\text{b}}=\frac{1}{N\left(N-1\right)}{\sum }_{i\ne \dot{J}{\epsilon}V}\frac{1}{{l}_{ij}}$$

where $V$ is the set of all nodes, and ${l}_{ij}$is the shortest path length of node $i$ and node $j$ (i.e., the path with the least number of edges).

b. Local efficiency

$$E\left(i\right)=\frac{1}{{N}_{Gi}\left({N}_{Gi}-1\right)}{\sum }_{\dot{J}\ne k{\epsilon}{G}_{i}}\frac{1}{{l}_{jk}}$$

$${E}_{loc}=\frac{1}{N}{\sum }_{i{\epsilon}V}E\left(\dot{i}\right)$$

where $E\left(i\right)$ represents the local efficiency of node $\dot{i}$, ${G}_{i}$ is the subgraph formed by the neighbors of node $\dot{i}$, and ${l}_{jk}$ denotes the shortest path length of node j and node $k$.

c. Degree.

Degree refers to the number of edges connected to a node. To analyze the constructed brain network and the neuromodulation effect of acupuncture, the node degree was selected for objective evaluation and analysis.

d. Betweenness centrality

$${N}_{bc}\left(i\right)={\sum }_{\dot{J}\ne i\ne k{\epsilon}G}\frac{{\sigma }_{jk\left(i\right)}}{{\sigma }_{jk}}$$

where ${\sigma }_{jk}$ denotes the number of shortest paths from node $\dot{J}$ to node $k$, and ${\sigma }_{jk\left(i\right)}$denotes the number of shortest paths that pass through node $\dot{i}$.

#### Secondary outcomes

Regarding secondary outcomes, the Wisconsin Card Sorting Test (WCST) will be used to assess cognitive function. The WCST is an evaluation tool used to measure cognitive abilities, such as abstract thinking, attention shifting, information memorization, and recognizing and responding to stimuli. It is highly sensitive to damage to the dorsolateral prefrontal cortex [18]. The PSQI will be used to assess sleep quality. It is an internationally recognized tool for assessing sleep quality that is suitable for use in patients with sleep disorders and in the general population [19, 20]. The timeline of each assessment is shown in Table 1.

**Table 1:** Participant timeline

Study Flow Chart						
Phase	Baseline period	Period of treatment		Period of follow-up		
Number of visits	1	2	3	4	5	6
Time (weeks)	Week 0	Week 4	Week 8	Week 12	Week 16	Week 20
Inclusion/exclusion criteria	**√**					
Informed consent obtained	**√**					
Demographic data	**√**					
Medical history and treatment history collected	**√**					
Pittsburgh Sleep Quality Index (PSQI)	**√**	**√**	**√**			**√**
Wisconsin Card Sorting Test (WCST)	**√**	**√**	**√**			**√**
EEG acquisition record sheet	**√**		**√**			**√**
Adverse event acupuncture record	**√**					
Combined medication records	**√**					
Adverse event (AE) recording form	**√**					
Serious Adverse Event (SAE) report form	**√**					
Treatment-Emergent Symptom Scale	**√**					
Assessment of adherence	**√**					
Study completion	**√**					
Review by investigators	**√**					

### Other measures

Researchers will identify any adverse effects through patient self-reports and monthly inquiries. All adverse events will be addressed, documented, and classified according to their relevance to acupuncture therapy. Researchers must inform the principal investigator and the Data and Safety Monitoring Board (DSMB) of serious adverse events within 24 h. Additionally, the subjects’ compliance and treatment side effects will also be recorded in the study.

### Data management

Case record forms will be used to collect and manage scale data, while EEG data will be securely stored on specialized hard drives monitored by the Data and Safety Supervision Committee. We will share the trial data before May 2025. The researchers can be contacted to obtain the data.

### Data availability statement

The minimum retention period for the data is 5 years after publication. The Principal Investigator will have access to the final test dataset, and the corresponding author can be contacted to access the test data. The patient’s identity, age, and phone number will remain confidential.

### Quality control

Experts in acupuncture and moxibustion, psychiatry, statistics and methodology reviewed and provided feedback on the test protocol. Before beginning the experiment, all researchers will be instructed on how to screen patients, administer the scales, perform acupuncture, and enter the data.

### Sample size

The primary outcome of this experiment is the PSQI score, and a two-sample t test for superiority by a margin will be adopted. Similar published studies have indicated that the mean PSQI scores of the EA and SA groups are 9.8 and 13.9, respectively, with standard deviations of 3.1 and 3.2. The SA group is expected to have a higher score than the EA group, with an expected score difference of 1.5. With a significance level of α = 0.025, power of 1-β = 0.9, and equal sample sizes of the SA and EA groups [21], the minimum sample size was calculated. According to the preset parameters, the “Two-Sample t Tests for Superiority by a Margin Allowing Unequal Variance” option in the Means menu of Power Analysis and Sample Size software (PASS; version 11; NCSS Statistical Software, Kaysville, UT, USA) was used for sample size calculation. A sample size of 32 was calculated for both groups. Considering a dropout rate of 20%, each of the three groups would require 40 participants. Therefore, 120 subjects will be included in the study to ensure the accuracy and scientific validity of the research results.

### Statistical analysis

In general, statistical involve a two-tailed approach, and any results that have a p value of less than 0.05 are considered statistically meaningful. Continuous data will be characterized by the mean ± standard deviation, median, highest value, and lowest value, while count and ordinal data will be represented via counts and percentages. The type of analysis used for between-group comparisons depends on the data distribution: paired-sample t tests or nonparametric tests will be used for continuous data, chi-square tests or Fisher’s exact test will be used count data, and nonparametric tests will be used for ordinal data. For comparisons of baseline values, paired-sample t tests or nonparametric tests will be used for continuous data, and nonparametric tests will be used for count data.

All EEG data will be processed using MATLAB (MathWorks, Inc., Natick, Massachusetts, USA). Graph theory analysis will be performed using the Gretna toolbox based on MATLAB. The analysis of brain network topological properties will employ graph theory methods. Specifically, the following steps will be used: segmentation according to the AAL template to divide the brain into 116 regions, computation of the average BOLD time series signals within each region, and calculation of Pearson correlation coefficients between each pair of regions. It will yield a 62 × 62 correlation matrix. The correlation matrix will then be converted into a binary matrix by applying a threshold r value (Pearson correlation coefficient) to construct a brain network. The topological properties of the brain network, including global efficiency, local efficiency, node degree, and node betweenness centrality, will be analyzed. Additionally, the relationships of topological properties with cognitive function and sleep quality scores will be examined through correlation analysis.

### Ethics and dissemination

The experimental protocol follows to the principles of the Declaration of Helsinki and has been approved by the Ethics Committee of the First Affiliated Hospital of Guangdong Pharmaceutical University (Medical Ethics Review [2021] No. 49). Modifications to the protocol will be reported. Researchers will receive instructions to provide trial information to qualified patients and obtain written informed consent if they agree to participate. The trial results will be published in peer-reviewed journals and made available to the media and the public.

## Discussion

Currently, the construction of functional brain networks mainly relies on techniques such as electroencephalography (EEG), magnetoencephalography (MEG), and functional magnetic resonance imaging (fMRI), combined with complex network analysis methods based on graph theory. These methods aim to reveal the topological principles of brain networks and the underlying mechanisms of brain function.

fMRI is an essential method for exploring brain networks, with the advantages of high spatial resolution and the ability to detect deep brain tissue activity. However, its temporal resolution is limited, resulting in a time delay between neuronal synchronization and the occurrence of relatively sharp wave discharges. DePasquale et al. [22] divided collected MEG data into 10-second time windows for separate data analysis, revealing significant differences in network connectivity within each time window. Different brain networks were active in various time windows, and several brain networks were sometimes activated simultaneously. This study suggested that fMRI research may overlook dynamic changes in brain networks due to its low temporal resolution. This highlights that recent fMRI-based brain network studies have focused on exploring static brain network topology within specific periods. Researchers have increasingly focused on constructing dynamic brain functional networks at short time scales, such as continuous time points, to better understand temporal changes and real-time brain activities.

Moreover, using different statistical approaches when processing fMRI data can lead to significant differences in research outcomes, raising questions about many past fMRI research conclusions [23, 24]. In general, brain functional network research using fMRI technology has become increasingly mature. However, its temporal resolution is still relatively low, and due to issues related to the choice of statistical methods, more evidence is needed regarding the use of fMRI to study brain networks.

Electroencephalography (EEG) is a noninvasive imaging technique that reflects brain electrical activity and functional states. It has high temporal resolution and provides quantitative information about the location and distribution of neural activity sources within the brain. EEG enables long-term, real-time monitoring of brain electrical activity and has the advantage of requiring specific hardware for recording and analysis. Despite its excellent temporal resolution, EEG has relatively low spatial resolution. The optimal approach is to use EEG inverse modeling to obtain activity information from the source space and then conduct brain network analysis based on this information. Therefore, the quest for a reliable research technique with high spatial and temporal resolution remains a current focus of research in this field. Combining electroencephalogram (EEG) with source localization analysis is a recently developed method of brain functional analysis that has attracted attention.

This is the first study to apply graph theory-based complex brain network analysis in combination with EEG and source localization methods to evaluate whether patients with chronic insomnia exhibit alterations in brain network topology attributes and to identify the influence of the dorsolateral prefrontal cortex, an important network node, in these changes. Additionally, a randomized controlled trial will be conducted to compare the changes in the topological attributes of the brain network of chronic insomnia patients from before to after electroacupuncture and to examine correlations of brain network parameters with scores on the Wisconsin Card Sorting Test (WCST) and the Pittsburgh Sleep Quality Index (PSQI) to identify the mechanism by which electroacupuncture enhances cognitive function and sleep quality in chronic insomnia patients.

We designed this trial according to the Good Clinical Practice guidelines, ensuring that it meets the methodological requirements for sufficient power, allocation concealment, and essential blinding [25]. According to our prior research, the acupoints chosen for this study are effective in treating insomnia.

This study has certain limitations. First, as acupuncture cannot be performed blindly, we will endeavor to minimize the impact by providing comprehensive training to the practitioners, which included standardized communication with the subjects. Second, we will not collect event-related potentials to evaluate specific cognitive functions. Third, the extended period of the study will likely lead some individuals to drop out.

### Trial status

This study was officially registered with the Chinese Clinical Trial Registry on 20 May 2022 with the registration ID ChiCTR2200060150. Recruitment of participants began on 1 September 2022 and is scheduled to be completed by the end of December 2024. In the event of any modifications to the protocol, we will inform the investigators, ethics committee, and trial registries.

## Data Availability

As stated in the informed consent form, the subject’s personal information will be kept private and not shared with the public unless the subject has given their consent. When needed, the ethics committee or project funding department can access the subject’s data for consultation. No other use of the subject’s information or disclosure of it to other groups is allowed without their permission.

## List of abbreviations

EEG: electroencephalography
DLPFC: dorsolateral prefrontal cortex
MPFC: medial prefrontal cortex
WCST: Wisconsin Card Sorting Test
PSQI: Pittsburgh Sleep Quality Index
DSMB: Data and Safety Monitoring Board
EA: electroacupuncture
SA: sham acupuncture
MEG: magnetoencephalography
fMRI: functional magnetic resonance imaging
